# Surgery vs. Medicine

**Published:** 1878-01

**Authors:** Wm. Tod Helmuth


### Surgery vs. Medicine.

BY WM. TOD HELMUTH, M. D.

Delivered at the banquet given at Delmonico's to the students and alumni of the New York Homœopathic Medical College, March 8, 1877.

I am a surgeon, and in making this assertion
'Tis my apology for doing what I can
To set aside that undeserved aspersion
That says, while medicine is quite as old as man,
Holding within its vast consideration
All wisdom, learning, ethics, and decorum,
That surgery is claimed, as is a poor relation,
Being at best "the *opprobrium medicorum*."

'Tis certainly a subject for humility,
And one 'tis hard *for doctors* to endure,
That they must own their utter inability
In many cases to effect a cure;
And then, with shrugs and sighs, their patients urge on
To give themselves the only chance for life
By calling on the poor, forgotten surgeon,
Who cuts and *cures* them with the dreaded knife.

But as for age, I'll prove it's all a libel,
(The statement's bold, but I could make it bolder),
For on no less authority than the Bible
I'll prove that surgery is surely older
Than any form of medicine whatsoever;
And having finished, will appeal to the majority
And have the point adjusted here forever,
That "*surgery in age* can claim priority."

'Tis true the snake aroused the curiosity,
And gave to Eve the apple fair and bright;
She ate, and with a fatal generosity
Inveigled Adam to a luscious bite.
That from that time disease and suffering came,
*Doctors* were called upon to cure the evil;
The art of *healing*, then, with all its fame,
Was *at the first* developed by the Devil.

Medicine thus stands coeval with the sinning
Of mother Eve, fair creature, though quite human,
While noble surgery had *its* beginning
*In Paradise*, BEFORE there was a woman.
The facts are patent, and we all agree
'Twas Satan laid on man the direful rod:
That *doctors* are the *Devil's progeny*,
While surgeons come *directly down from God!*

For thus we read (although the analgesia
Of Richardson was then entirely unknown),
Adam profoundly slept with anæsthesia,
And from *his thorax was removed a bone.*
This was the first recorded operation,
(No doctor here dare tell me that I fib!)
And surgery, thus early in creation,
*Can claim complete excision of a rib!*

But this is nothing to the obligation
The world to surgery must ever own,
When woman, lovliest of creation,
Grew and developed from *that very bone.*
Then lovesick swains began inditing sonnets,
And Fashion talked with Folly by the way,
Then came boulimia for becoming bonnets—
Hereditary epidemic of to-day.

Then, too, began those endless loves and frolics
That poets sang in soft and sweet refrains,
Doctors grew frautic o'er infantile colics,
Announced at midnight with angelic strains.
From this the world was peopled. So you must own,
While you lay claim to such superiority,
That surgery, in the development of bone
As well as age, can clearly claim priority.

My task is done, and with my best endeavor
I have essayed to vindicate my art;
So list, my friends, ere friendly ties we sever,
While waning moments bring the hour to part:
Whatever land, whatever clime may hold you,
Some time give honor to the bright scalpel,
And when you recollect what I have told you,
Remember me—'tis all I ask. Farewell.

### SMALL FRUIT IN GARDENS.

But few people seem to know the value of small fruits to a family, when grown in their own gardens. You commence with strawberries; they continue about a month. You pick, perhaps, from six to twelve quarts a day. You have them on your table as a desert, if you please at noon, and your table is loaded with them at evening, and you want little else but your bread and butter.—Your family consume in one way or another, about eight quarts a day, and while they last no medicines for bodily ailments are required, as a quart of strawberries daily will generally dispell all ordinary diseases not settled permanently in the system. After strawberries, raspberries come to continue about three weeks; then we have blackberries where the climate is not too cold for cultivated varieties; then currants ripen, which remain until the early grapes ripen; and taking the season through any family with a half acre of land in a garden can grow small fruits that make country life delightful, and at the same time hundreds of dollars can be saved in the supply of the table.—*Chautauqua Farmer*.

### GRANULATED WHITE SUGAR.

Mr. E. W. Runyon had found, on comparison, that the several brands of supposed A No. 1 granulated sugar of the market, produced syrups of different tints, which suggested an examination of their quality. Samples were found containing considerable proportionof ultramarine, which after several days' standing, were deposited. Syrups made from sugars having the ultramarine impurity are discolored, being usually of a pale straw color.

This adulteration and additions of sulphate of tin, alum, etc., are used by refiners in the interest of dollars and cents, and are designed to neuteralize the yellow tint in imperfectly refined sugars.—The practice is known among the refiners as adding the complementary color.

Unquestionably ultramarine adulteration is chemically injurious, being decomposed by fruit or organic acids with evolution of sulphuretted hydrogen, which produces a disagreeable taste; aside from this serious objection the officinal syrups instead of being colorless and bright are tinted and dull in appearance.

Pure sugars can be had by purchasing from first-class manufacturers and paying a slight advance on the price of ordinary marketabte granulated white sugar.

---

The famine in China still continues to prevail. The scarcity of food has been so great that only human beings remain, and they have been obliged to subsist upon the bark of trees, coarse mosses, and some have even eaten the thatch of their houses.

---

There is great destitution among those who went to the Black Hills with such glowing anticipations. Recently twelve men offered to draw a loaded wagon out of the country if they could be fed on the road.

